# Acrylamide acute neurotoxicity in adult zebrafish

**DOI:** 10.1038/s41598-018-26343-2

**Published:** 2018-05-21

**Authors:** Melissa Faria, Tamar Ziv, Cristian Gómez-Canela, Shani Ben-Lulu, Eva Prats, Karen Adriana Novoa-Luna, Arie Admon, Benjamin Piña, Romà Tauler, Leobardo Manuel Gómez-Oliván, Demetrio Raldúa

**Affiliations:** 1 IDAEA-CSIC, Jordi Girona 18, 08034 Barcelona, Spain; 2 Technion – Israel Institute of Technology, The Smoler Proteomics Center and the Department of Biology, Haifa, Israel; 3 CID-CSIC, Jordi Girona 18, 08034 Barcelona, Spain; 4 Laboratorio de Toxicología Ambiental, Facultad de Química, Universidad Autónoma del Estado de México, Paseo Colón intersección Paseo Tollocan s/n. Col. Residencial Colón, 50120 Toluca, Estado de México Mexico

## Abstract

Acute exposure to acrylamide (ACR), a type-2 alkene, may lead to a ataxia, skeletal muscles weakness and numbness of the extremities in human and laboratory animals. In the present manuscript, ACR acute neurotoxicity has been characterized in adult zebrafish, a vertebrate model increasingly used in human neuropharmacology and toxicology research. At behavioral level, ACR-treated animals exhibited “depression-like” phenotype comorbid with anxiety behavior. At transcriptional level, ACR induced down-regulation of regeneration-associated genes and up-regulation of oligodendrocytes and reactive astrocytes markers, altering also the expression of genes involved in the presynaptic vesicle cycling. ACR induced also significant changes in zebrafish brain proteome and formed adducts with selected cysteine residues of specific proteins, some of them essential for the presynaptic function. Finally, the metabolomics analysis shows a depletion in the monoamine neurotransmitters, consistent with the comorbid depression and anxiety disorder, in the brain of the exposed fish.

## Introduction

Acrylamide (ACR) is a water-soluble alkene widely used in the paper and textile industries, as flocculant in the wastewater treatment and municipal drinking water, as soil conditioner, as chemical grout in tunnels, sewers and wells, in ore processing, and in cosmetics^1,2^. Reports of ACR poisoning after occupational or accidental exposure to ACR indicated major symptoms related to polyneuropathy, including lethargy, ataxia, skeletal muscle weakness and numbness of the extremities^3–5^. Although early studies suggested that ACR neurotoxicity was associated with central-peripheral distal axonopathy^6^, it is currently well-established that the molecular initiating event (MIE) of ACR neurotoxicity is the disruption of presynaptic vesicle cycling by selectively forming adducts with thiolate sites located on proteins specifically involved in vesicle docking (synaptotagmin, synaptophysin, and syntaxin), vesicle priming (complexin-2), SNARE core dissolution (N-ethylmaleimide sensitive factor), endocytosis (clathrin), neurotransmitter re-uptake (membrane dopamine transporter) and vesicular storage (vesicular monoamine transporter) at the nerve terminals^7^. Consistently with this adverse effect of ACR on the presynaptic terminals function, a decrease in the monoamine neurotransmitters serotonin, norepinephrine and dopamine content in rat brain has been reported^8,9^. As monoamine depletion is believed to result in depression and anxiety^10^, the potential of ACR to induce these psychological disorders should be conveniently addressed.

Zebrafish (*Danio rerio*) is a vertebrate model increasingly used in biomedical research, including neurotoxicology studies^11–16^, as this animal species exhibits a similar overall nervous system organization to humans and similar neurotransmitter systems, including glutaminergic, cholinergic, serotonergic, dopaminergic, adrenergic, GABAergic, and histaminergic^11,15,17^. Recently we characterized the ACR acute neurotoxicity in zebrafish larvae by exposing 5 days post-fertilization (dpf) animals to 1 mM ACR for 3 days^15^. The reported results suggest that the adverse outcome pathways behind the ACR acute neurotoxicity in zebrafish larvae are similar to those described in humans and mammalian models^15^. However, adult zebrafish is more feasible than larvae for modeling complex brain disorders due to their well-developed central nervous system (CNS) and more complex behaviors^18,19^. Moreover, as zebrafish are a less sentient species, the use of adult zebrafish for modeling brain disorders fully meets the 3Rs principles (Replacement, Reduction and Refinement)^18^. Adult zebrafish have been used to model affective disorders, including stress-related, anxiety spectrum, depression, post-traumatic and phobic disorders, and multiple behavioral paradigms, resembling well-established rodent tests, have been developed^18,20^.

In this study, we characterized ACR acute neurotoxicity in adult zebrafish by waterborne exposure of the animals to 0.75 mM ACR for 3 days. Behavioral effects have been thoroughly analyzed, and changes in the transcriptomic, proteomic and neurochemical profiles in the brain of the exposed fish have been determined. Finally, the presence of ACR adducts in selected cysteine residues of specific brain proteins has been analyzed.

## Results and Discussion

### Systemic toxicity

The preferred concentration to be used in this study should be high enough to maximize the chance of detecting a neurotoxic effect, but not so high to induce systemic toxicity, an important confounding factor^21^. In mammalian toxicology this concentration at the threshold of the systemic toxicity is known as Maximum Tolerated Dose (MTD), and this concept has been translated to fish toxicology as Maximum Tolerated Concentration (MTC)^22^. Therefore, a total of 123 adult zebrafish were exposed for 72 h to different ACR concentrations and mortality was recorded. Whereas the 50% lethal concentration (72h-LC50) was estimated at 1.22 ± 0.02 mM ACR, the non-observed effect concentration (NOEC) for lethality was 0,75 mM ACR (Supplemental Fig. S1).

### Behavioral effects

**Figure 1:**
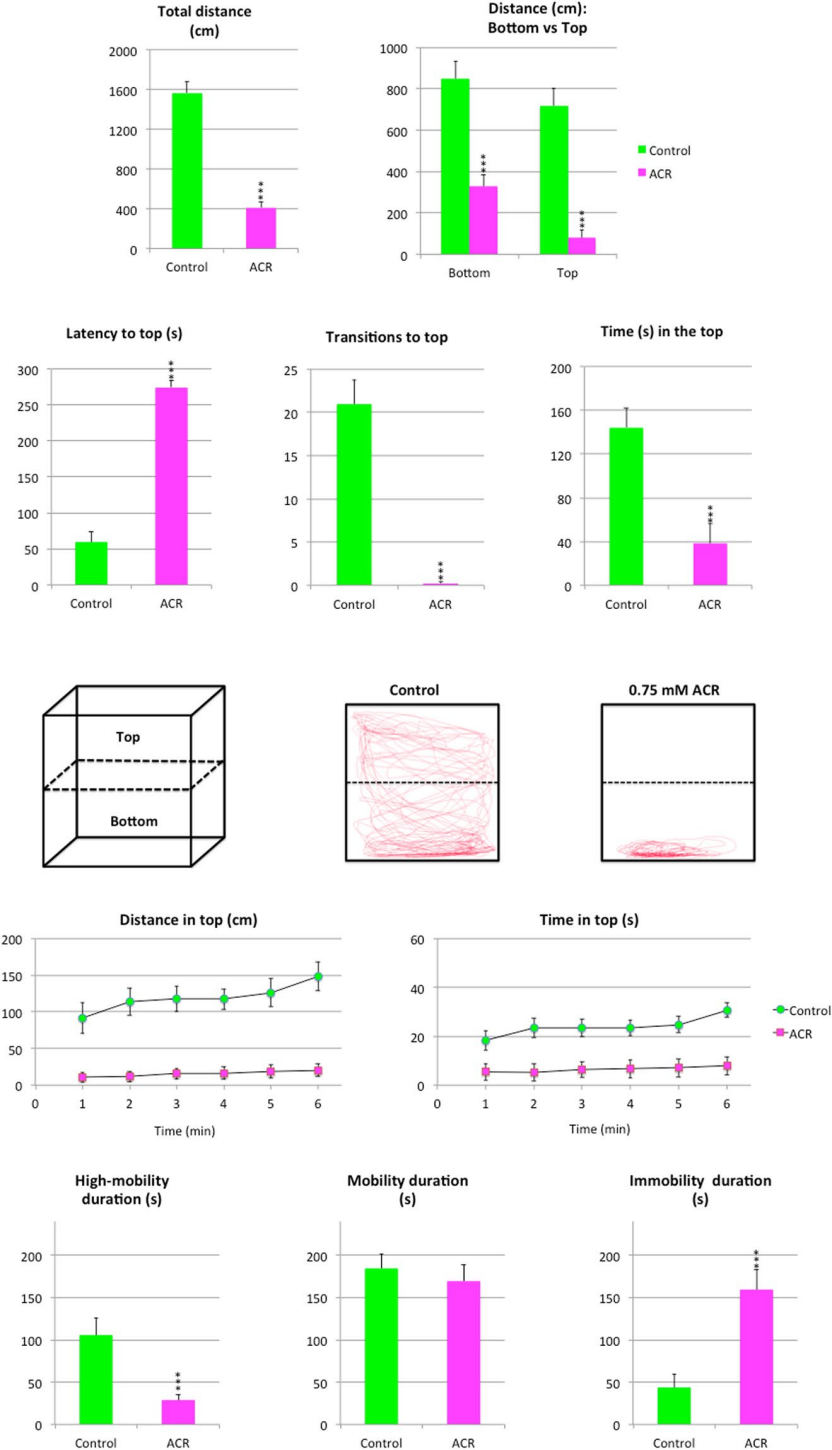
Behavioral effects of 3 days exposure to 0.75 mM acrylamide (ACR) on zebrafish tested in the novel tank. (**a**) Behavioral parameters assessed in standard 6-min novel tank test (NTT), as well as a cartoon of the experimental tank divided into two equal virtual zones, top and bottom and representative traces of control and ACR-treated zebrafish.

**Figure 2:**
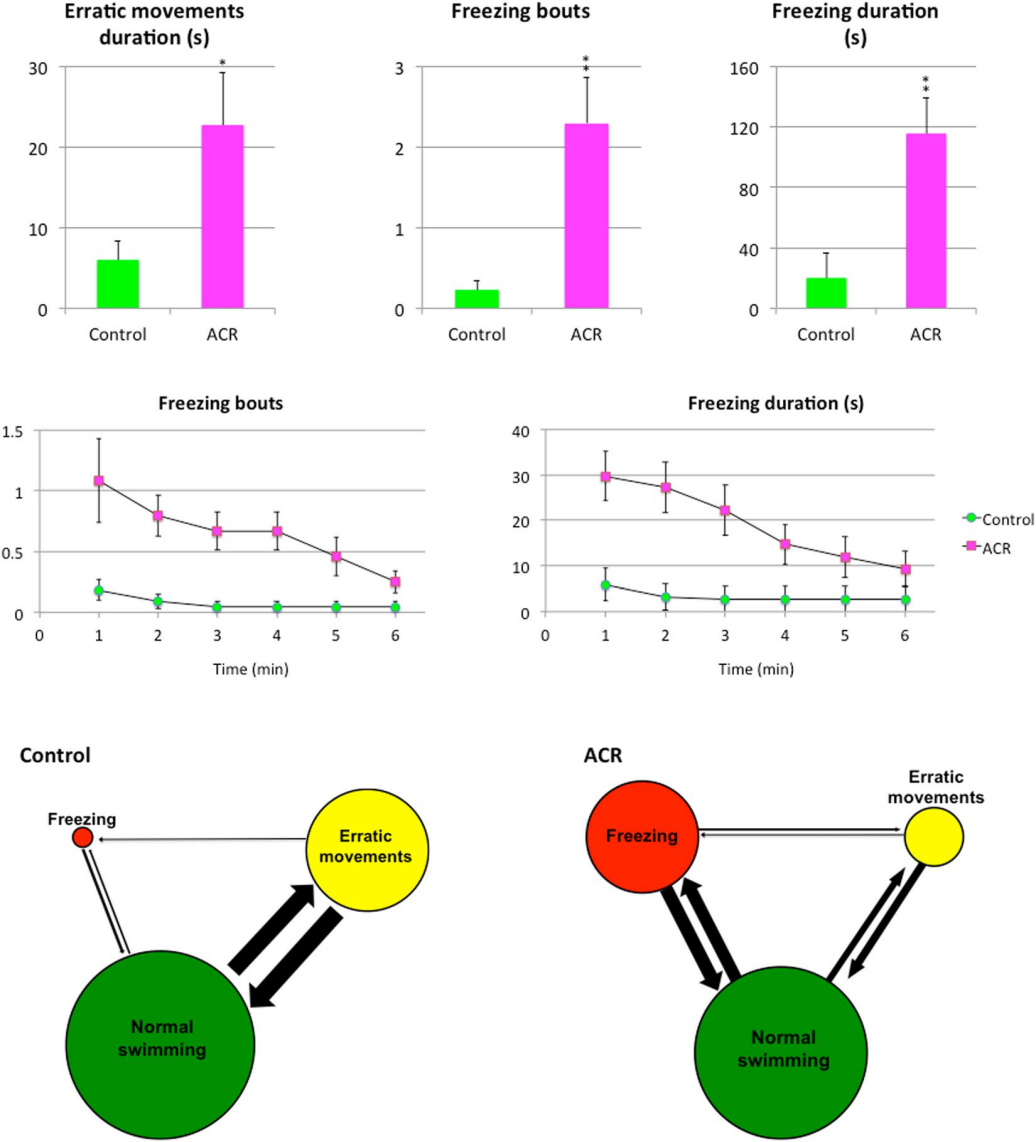
Behavioral effects of 3 days exposure to 0.75 mM acrylamide (ACR) on zebrafish: erratic movements and freezing bouts. Erratic movements, number and duration (s) of freezing bouts manual and ethograms allowing to visualize the occurrence of these behaviors and the transitions between them, with the diameter of each circle reflecting the frequency of the behavioral activity, and the width and direction of each arrow representing the frequency of the transitions between behaviors. Data reported as mean ± SEM (n = 23–24), *p < 0.05, **p < 0.01, ***p < 0.001 (Student’s t-test). Data from 3 independent experiments.

Results of the Novel Tank Test (NTT) with control and ACR-treated fish are shown in Figs 1 and 2. First of all, ACR induced hypolocomotion in the NTT, with a significant decrease in the total distance travelled (p < 0.001). The reduction observed in the distance travelled was more dramatic in the top of the tank than in the bottom (Supplementary Video S1). Moreover, ACR treatment increased the latency to top, transitions to top and time in top (p < 0.001). Whereas ACR treatment increased the latency to top, transitions to top and time in top were significantly reduced. Representative traces generated by Ethovision XT 13.0 software clearly support the dramatic effect of ACR on the top swimming (Fig. 1). The time-course analysis of the distance moved in the top and the time in top shows that control fish progressively increase the activity in the top of the tank with time, a typical habituation response to a novel environment^23,24^. In contrast, ACR-treated fish exhibited a very low activity in the top throughout the 6 min period. ACR also altered the mobility state duration of the fish, significantly decreasing the high-mobility duration (p < 0.001) and increasing the immobility duration (p < 0.001). ACR significantly increased also the number of freezing bouts (Supplementary Video S2; p < 0.01), the freezing duration (p < 0.01) and the erratic movements duration (p < 0.05). Moreover, ACR increased the transitions from freezing to normal swimming, from freezing to erratic and from normal swimming to freezing. Ethogram-based analyses provided another intuitive way to assess the changes in activity observed in ACR-treated fish (Fig. 2). Finally, ACR evoked a clear droopy tail phenotype (Supplementary Video S3), commonly associated with neurological deficits, akinesia and global hypolocomotion, in 21% of the treated fish. The prevalence of this phenotype in the control group was 0%.

**Figure 3:**
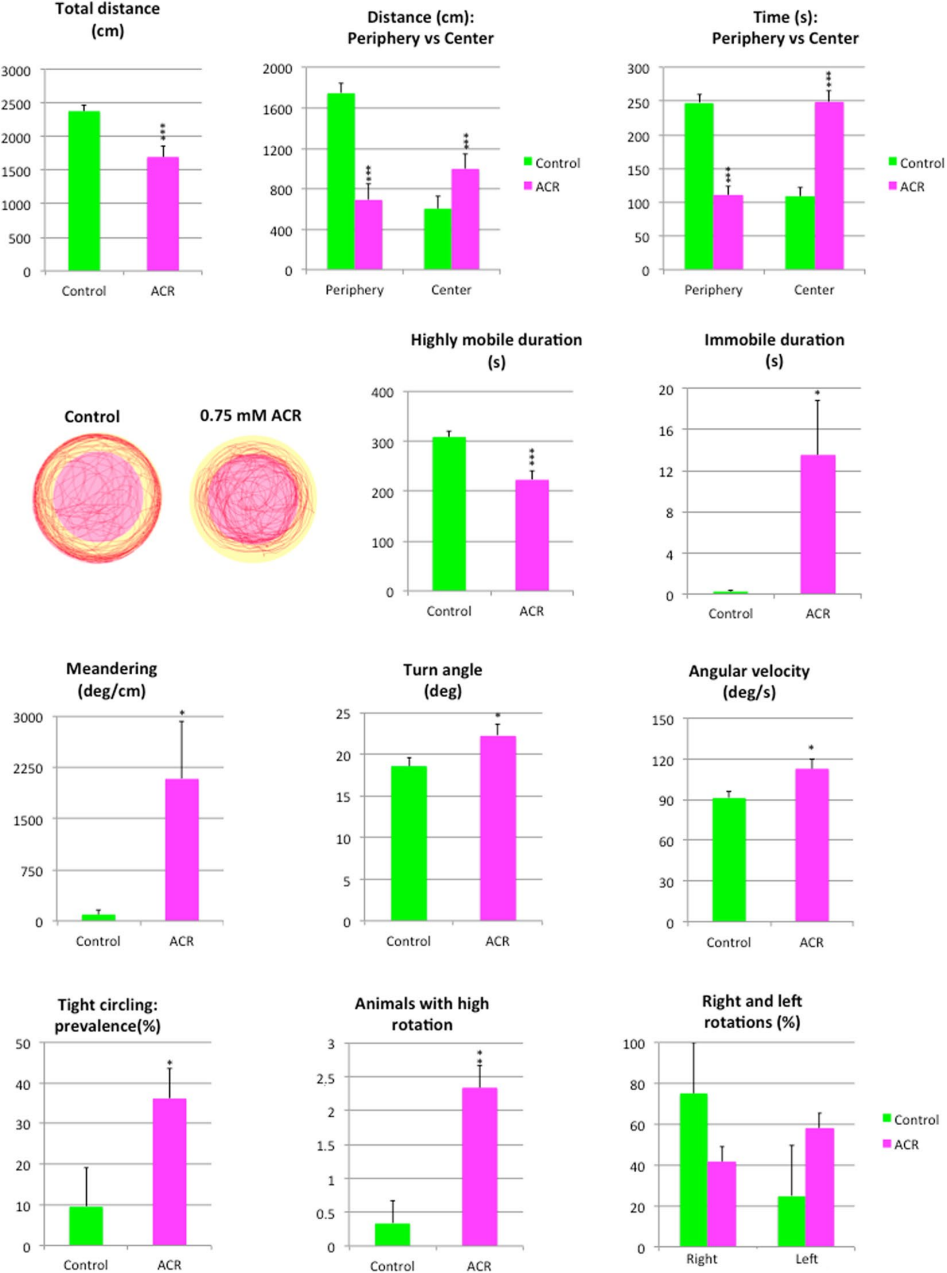
Behavioral effects of 3 days exposure to 0.75 mM acrylamide in the open field. Behavioral parameters assessed in standard open field test (OFT), as well as representative traces of control and ACR-treated zebrafish, showing the two virtual zones, center and periphery, in the arena.

Results of the Open Field Test (OFT) performed on control and ACR-treated zebrafish are shown in Fig. 3. Similarly to the NTT results, ACR induced hypolocomotion in zebrafish, with a significant reduction (p < 0.001) in the total distance moved in the tank during the 6 min period of the test. However, when the distribution of the locomotor activity between the center and the periphery of the tank was analyzed, a negative thigmotaxis was found in the ACR-treated fish. Thus, whereas the distance and time in the periphery were significantly reduced (p < 0.001), a concomitant increase was found both in the distance moved and the time spent in this central zone (p < 0.001). Representative traces generated by Ethovision XT13.0 software clearly support the negative thigmotaxis induced by ACR (Fig. 3). Changes observed in the mobility state duration were consistent with those found in the NTT, with a significant decrease in the high mobility duration (p < 0.001) and an increase in the immobility duration (p < 0.05). When parameters related with changes in direction of the movement were analyzed, a significant increase in the absolute meandering, turn angle and angular velocity (p < 0.05) were found in ACR-treated fish. Moreover, a tight circling behavior^25^, with repetitive swimming in circles of two body-lengths (about 5 cm) in diameter was identified in the ACR-treated group (Supplementary Video S4), with a significant increase in the prevalence of this behavior (p < 0.05) and in the number of animals exhibiting high rotation (p < 0.01), defined as 5 or more full rotations per trial^26^. No lateralization in the direction of rotations (p > 0.05) was found in any experimental group.

**Figure 4:**
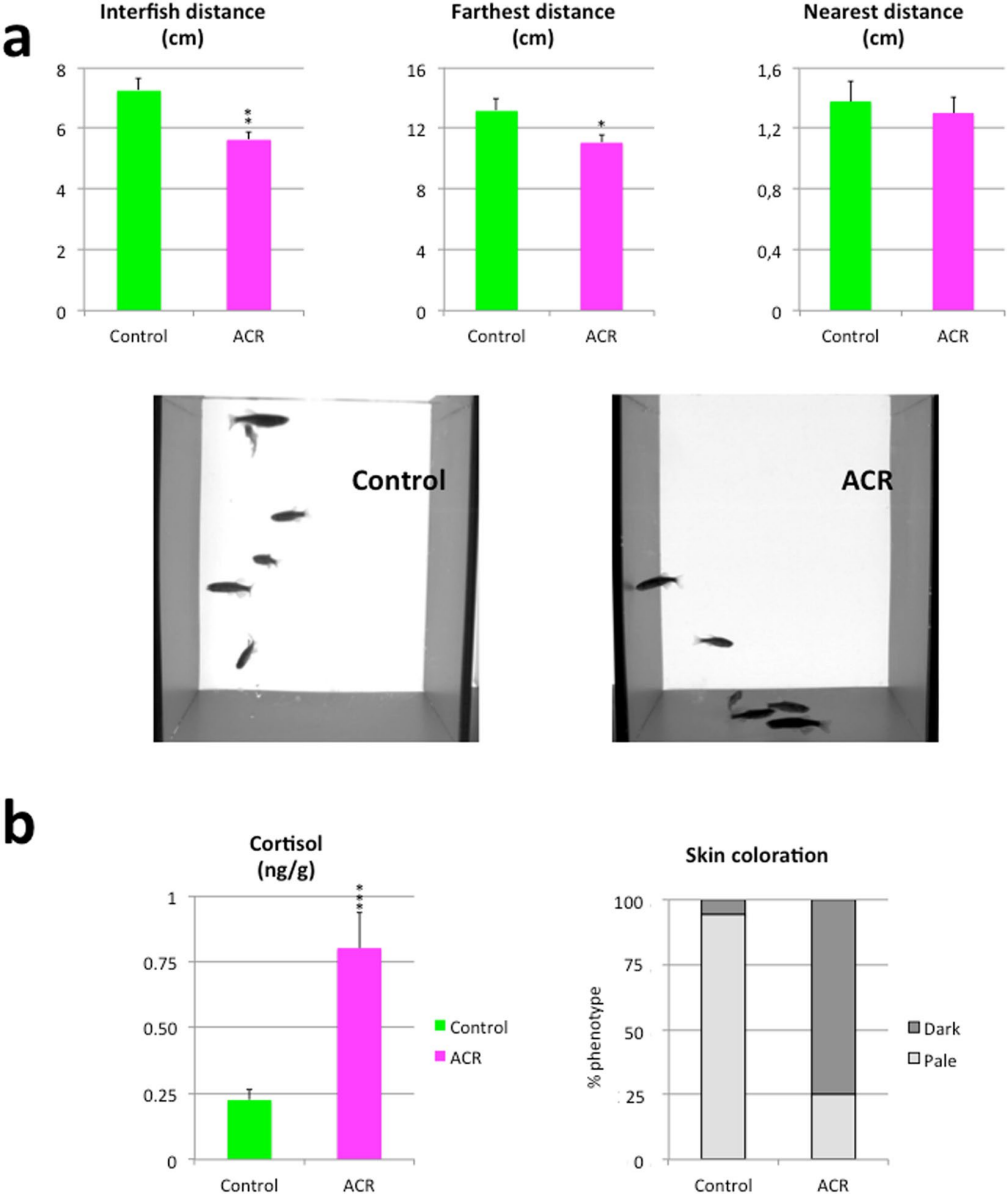
Effects of 3 days exposure to 0.75 mM acrylamide on the shoaling test, cortisol levels and skin coloration. (**a**) Behavioral parameters of zebrafish shoaling behavior in control and ACR-treated zebrafish. Data reported as mean ± SEM (n = 23–25 for OFT and n = 16–18 for shoaling test), *p < 0.05, **p < 0.01, ***p < 0.001 (Student’s t-test). Data from 3 independent experiments. (**b**) Whole-body cortisol levels and skin coloration in control (n = 16–18) and ACR-treated (n = 14–16) adult zebrafish. Data from 3 independent experiments.

Results from the shoaling test with control and ACR-treated fish are shown in Fig. 4a. ACR significantly increases social cohesion, as indicated by the significant decrease in the inter-fish distance (p < 0.01) and farthest distance (p < 0.05).

When the whole-body cortisol levels were analyzed, a significant increase was found in those animals exposed to ACR [Fig. 4b; 0.226 ± 0.041 vs 0.802 ± 0.135 ng/g for control (n = 16) and ACR-treated (n = 14) zebrafish, respectively; p < 0.0005]. Finally, a consistent darkening of the zebrafish skin coloration was found in the ACR-exposed group [Fig. 4b; χ^2^ (1) = 17.298, p < 0.001].

ACR neurotoxicity in humans and mammalian animal models is characterized by an impairment in the motor function^7^. Hypolocomotion observed in the NTT and OFT in the ACR-treated zebrafish in this study is consistent with the reduction in the basal locomotor activity found in the zebrafish model developed in larvae^15^. In zebrafish, hypolocomotion and droopy tail phenotype are indicative of motor retardation, a common motor symptom of clinical depression^25,26^. Moreover, elevated corticosteroids are also commonly seen in depressed patients and in animal experimental models of depression^27^. Thus, hypolocomotion, droopy tail and the significant increase in the whole-body cortisol levels found in the ACR-exposed animals are all hallmarks of a depression-like phenotype. Finally, the changes in the skin coloration presented by the ACR-exposed zebrafish are similar to the long-term skin darkening reported in zebrafish exposed to the catecholamine-depleting drug reserpine^28^, an additional data supporting the induction of a depression-like phenotype by ACR exposure. However, not all the clinical symptoms found in the ACR-exposed fish are consistent with a depressive disorder. Thus, some of the most significant behavioral changes exhibited by ACR-treated fish in the NTT (increase in the bottom dwelling behavior, the number of freezing bouts, freezing duration and erratic movements) and in the shoaling test (increase observed in the shoal cohesion) are typical responses to anxiogenic stimuli in adult zebrafish^29–31^.

Although depression and anxiety have historically been seen as distinct conditions, the two disorders are frequently comorbid, with approximately two thirds of patients with depression have a comorbid anxiety disorder^32^. The behavioral results presented here demonstrate that acute exposure to ACR induces depression-like comorbid with anxiety in adult zebrafish.

### Effects on transcriptional level

**Figure 5:**
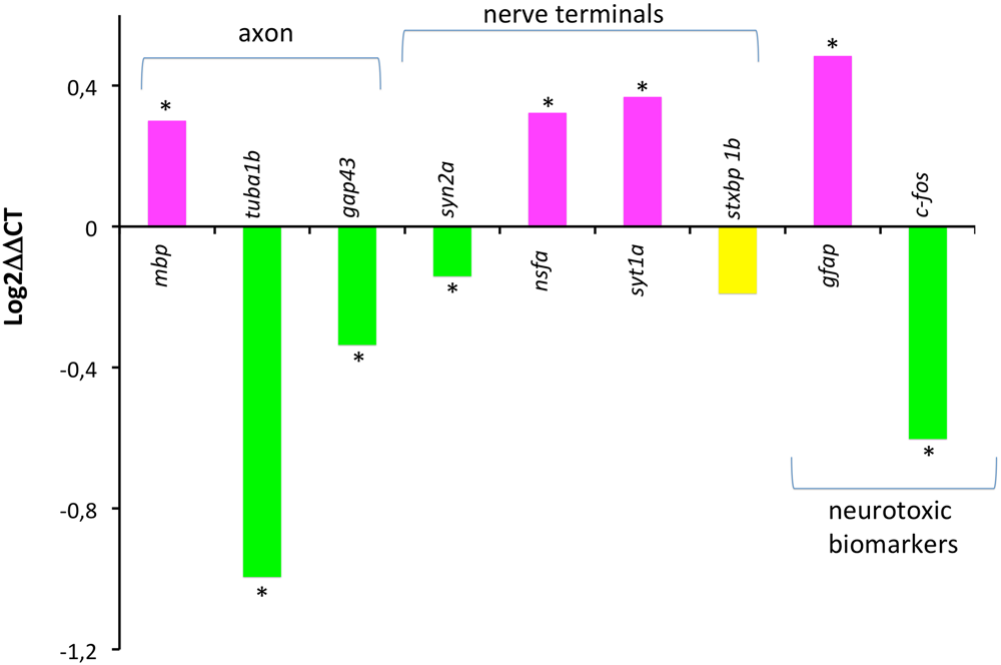
Expression of selected transcripts related with axonal damage, synaptic vesicle cycling and neurotoxicity in the brain of adult zebrafish exposed to 0.75 mM acrylamide (ACR) for 3 days. Results are plotted as log2 ΔΔCt, in order to see both the up regulation and the down regulation. *p < 0.05 (Student’s t-test). Data from 3 independent experiments (n = 14–17).

In order to better characterize the ACR targets in the adult zebrafish CNS, the expression of genes involved in different functions in neurons and glia was analyzed (Fig. 5). First of all, at the axonal level, a significant down-regulation was found in the expression of the regeneration-associated genes (RAGs) growth-associated protein GAP-43 (*gap43*) and α1 tubulin (*tuba1b*). The expression of myelin basic protein (*mbp*) and glial fibrillary basic protein (*gfap*), expressed in oligodendrocytes and reactive astrocytes, was found up-regulated. Moreover, ACR induced changes in the expression of genes involved in the synaptic vesicle cycling. Thus, whereas the expression of *nsfa* and *syt1a* were found up-regulated, *syn2a* expression was down-regulated. Finally, the expression of the immediate early gene *c-fos* was down-regulated.

In teleosts, including zebrafish, the success of CNS regeneration may be determined, at least in part, by the induction of RAGs^33^. The absence of this regenerative response in ACR-treated zebrafish, as indicated by the significant down-regulation in the expression of *gap43* and *tuba1b* in this study and by the absence of induction of these genes in zebrafish larvae^15^, is consistent with the absence of severe axonal damage reported in acute ACR exposures^7^. Moreover, the up-regulation of *gfap* found in the brain of ACR-treated zebrafish in this study is consistent with the increase in the GFAP immunoreactivity reported in the brain of rats and mice exposed to ACR^34,35^. However, this effect on *gfap* expression was not found in ACR-treated zebrafish larvae^15^, probably reflecting differences in the regulation of this gene with the developmental stage. The up-regulation found in the synaptic vesicle cycling genes *nsfa* and *syt1a* in the brain of ACR-treated zebrafish paralleled the effect of this compound in zebrafish larvae^15^. The finding of a similar effect of ACR on the expression of *nsfa* and *syt1a* in zebrafish larvae (immature central and peripheral nervous system), and the adult brain (mature CNS), makes these two genes good candidates as ACR neurotoxicity markers. Finally, *c-fos* expression is a marker of neuronal activation in rodents and zebrafish, and has been shown to correlate with environmentally or pharmacologically induced anxiety^36^. Thus, the down-regulation of *c-fos* found in this study in response to ACR strongly supports that the behavioral effects observed in ACR-treated zebrafish are not related to an increase in the anxiety levels.

### Effects of ACR on the adult fish brain proteome

**Figure 6:**
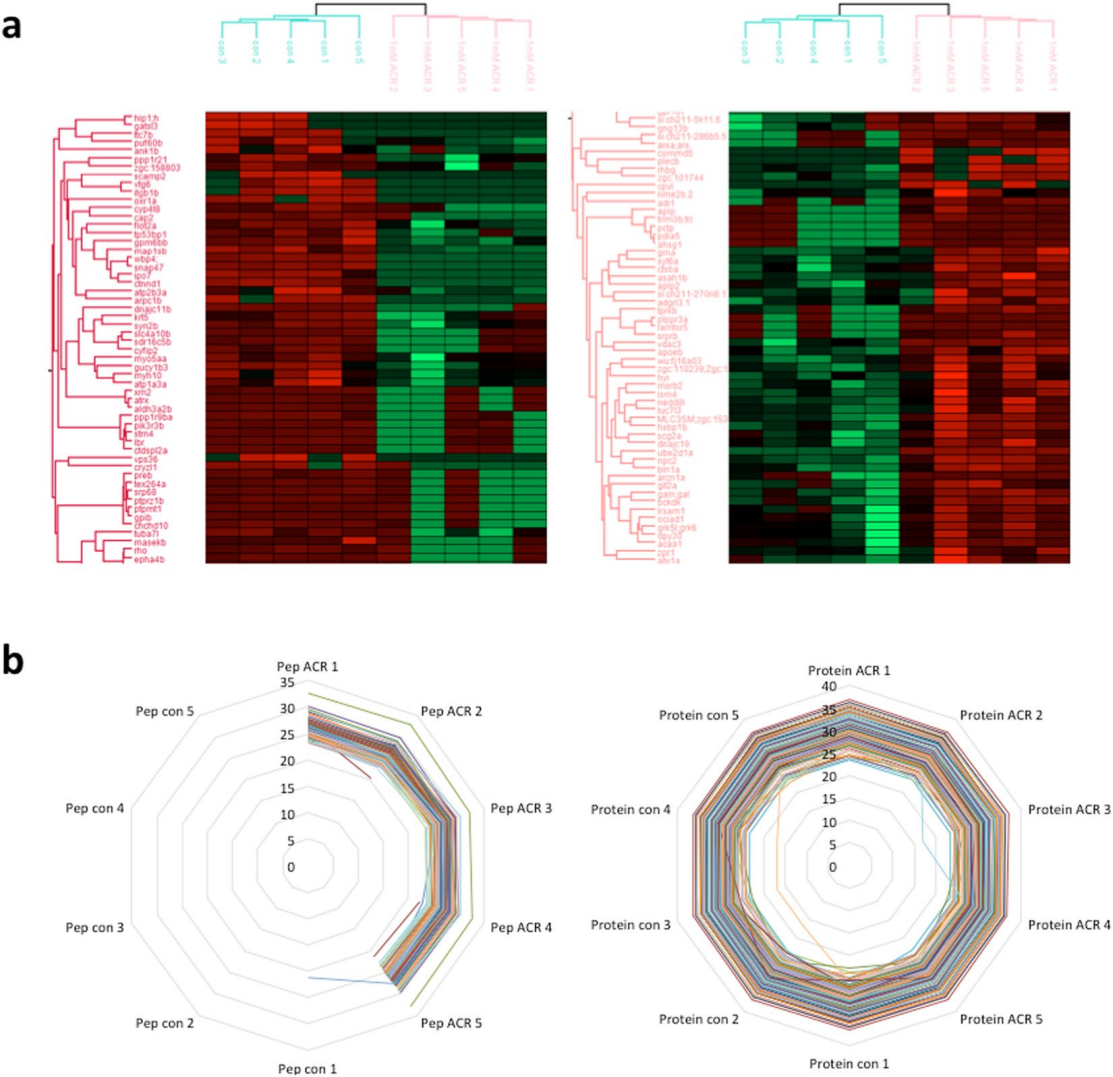
Effects of acrylamide exposure on the adult zebrafish brain proteome. (**a**) Unsupervised heat map of the differential proteins. Hierarchical euclidean clustering was performed on the intensities of the differential proteins after Z-score normalization. It was done using the Perseus software. Left panel represents the down-regulated proteins and the right panel the up-regulated proteins following the ACR treatment. (**b**) ACR-modified cysteine residues. Left panel represent the expression levels of the 191 proteins containing ACR-modified cysteine residues, and the right panel the Log-2 intensities of the peptides with ACR –adducts in cysteine residues. CN: control pool (n = 5 pools; 3 brains each); ACR: pool ACR (n = 5 pools; 3 brains each).

The current view is that ACR induce synaptotoxicity in mammals and that the MIE is the formation of ACR-adducts in cysteine residues involved in the synaptic vesicle cycle at the nerve terminal, resulting in altered function of the modified proteins and, finally, in altered neurotransmission^7^. In order to determine if ACR altered the expression of proteins involved in synaptic vesicle cycle in the nerve terminal of adult zebrafish brain, the proteome of control and exposed animals was compared. Five pools of 3 brains from either ACR-treated or untreated groups were proteolyzed and analyzed by capillary chromatography and tandem mass spectrometry (μLC-MS/MS). A total of 4,515 proteins were identified in at least 3 of the samples and with at least 2 different tryptic peptides (Supplementary Dataset 1). Following the ACR treatment the intensities of 57 proteins were down-regulated while the intensities of 65 proteins were up-regulated (Fig. 6a and Supplementary Dataset 2).

One interesting protein up-regulated after ACR treatment was Adhesion G protein-coupled receptor L3.1 (Adgrl3.1), involved in ADHD and addiction in humans^37^. This protein has been suggested to modulate dopaminergic neuron formation and locomotor activity during zebrafish development^37^. Moreover, loss of function of Adgrl3.1 causes an hyperactive motor phenotype in both zebrafish^37^ and mice^38^, and this phenotype can be rescued by ADHD treatment drugs^37^. Granulin a, the homologue of human GRN based in syntenia^39^, and Synuclein gamma b, were also up-regulated in the brain of ACR-treated zebrafish. Up-regulation of progranulin, the precursor of Granulin a, seems to be part of the inflammatory response after acute or chronic insults in the CNS in mammalian models^40^. Synuclein gamma b up-regulation, hypolocomotion and an increase in freezing bouts and freezing duration in the NTT, some of the effects of acute exposure to ACR identified in this study, were also found in a model of Parkinson’s disease in zebrafish induced by 1-metyl-4-phenyl-1,2,3,6-tetrahydropyridine (MPTP)^41^. Finally, the peptide Galanin was also found up-regulated in the brain of ACR-treated animals. Hypolocomotion and depression-like behavior have been also described in mice overexpressing galanin^42^. Similar to the transcriptomic results, proteomic analysis found down-regulation of Tubulin alpha chain and Synapsin IIa. Down-regulation was also found in another two proteins involved in neurotransmission, Huntingtin interacting protein 1 (Hip1) and Synaptosomal-associated protein 47 (Snap47). Whereas Hip1 plays a role in clathrin-mediated endocytosis and trafficking, Snap47 is involved in regulating AMPA receptor trafficking in the CNS in an NMDA-dependent manner^43,44^.

Sulfhydryl thiolate state of cysteine residues are the preferred target of for the covalent interactions of ACR^7^. At physiological conditions, sulfhydryl thiolate groups can be found in cysteine-centered catalytic triads, often located within the active sites of many critical presynaptic terminal enzymes. ACR adducts formed with the cysteine located at the catalytic triads will have substantial implications for protein function and subsequent presynaptic toxicity^31^. When the presence of covalent acrylamide adducts was analyzed in control and ACR-exposed larvae, 191 proteins were detected with modifications on specific cysteine residues in at least 3 of the ACR-pools (Fig. 6b and Supplementary Dataset 3). Fifty-three of these modified proteins were also modified in the larva stage as was shown before, suggesting developmental conservation of the target proteins (Supplementary Dataset 4). Interestingly, some proteins specifically involved in the synaptic vesicle cycle, were modified by ACR. Thus, ACR formed adducts at Cyst91 and Cyst106 of Complexin 2, a cytosolic protein that positively regulates a late step in synaptic vesicle exocytosis^45^. ACR adducts were also found at Cyst155 and Cyst417 of Synapsin I and IIb, respectively. Synapsins are phosphoproteins binding synaptic vesicles to components of the cytoskeleton which prevents them from migrating to the presynaptic membrane and release their neurotransmitters^45^. Synaptotagmin VIIa, calcium sensor playing a crucial role in neurotransmitter release, was found altered by ACR at Cyst 413, 431 and 532. Finally, ACR formed adducts at Cyst142 of vesicle-associated membrane protein (VAMP). Whereas VAMP/synaptobrevin forms together with SNAP-25 and syntaxin the presynaptic SNARE complex, VAPA interacts with VAMP and is necessary for vesicular neurotransmission^45^. Some protein related to the dendritic spines, as Arp2/3 complex 34 kDa subunit, Debrin 1 and Striatin calmodulin-binding protein, were also modified by ACR.

Other enriched groups of proteins that were found to be modified by ACR adducts include processes of cell redox homeostasis (Thioredoxin, Thioredoxin 2, Peroxiredoxin 2, Peroxiredoxin 6), as well as proteins involved in biosynthesis of amino acids and glycolysis/gluconeogenesis (Fructose-bisphosphate aldolase, Glutamine synthetase, Aspartate aminotransferase, Phosphoglycerate kinase, Phosphoglycerate mutase). Interestingly, many of the synaptosomal proteins known to be modified by ACR in rat brain^46^, including Complexin 2, Synaptotagmin, Secernin 1, Ubiquitin carboxyterminal hydrolase isozyme L1, Glutamine synthetase 1, Cofilin-1, Voltage-dependent anion-selective channel protein 3, Aspartate aminotransferase, and Ubiquinol-cytochrome c reductase complex, were also found modified in the zebrafish brain.

The expression levels of most proteins were not changed as a result of the modification, with the exception of COX17 cytochrome c oxidase assembly homolog, Glycoprotein M6Bb, Hydroxypyruvate isomerase, Tubulin alpha chain, Synapsin II and Voltage-dependent anion channel 3 (Fragment). It is very interesting that both Synapsin II and Tubulin alpha chain, significantly down-regulated at the transcriptome and the proteome levels, were found to be modified by the ACR both in the brain as well as in the whole-larvae samples.

### Changes in the neurochemical profile

In order to assess if ACR altered neurotransmission in adult fish brain, thirty-eight neurochemicals, including neurotransmitters, precursors, metabolites and neuromodulators, were analyzed in individual brains from the control (n = 12) and ACR-treated (n = 12) groups, from 3 independent experiments. Prior to the collection of the brain samples, behavioral analysis of each animal was performed through the NTT and OFT, in order to be able to associate to each fish both a neurochemical and neurobehavioral profile. Thirty-six of the neurochemicals analyzed in the brain of fish from both experimental groups were above the limit of quantification (Supplementary Table S2), and the level of seventeen of them was significantly altered by the ACR-treatment.

**Figure 7:**
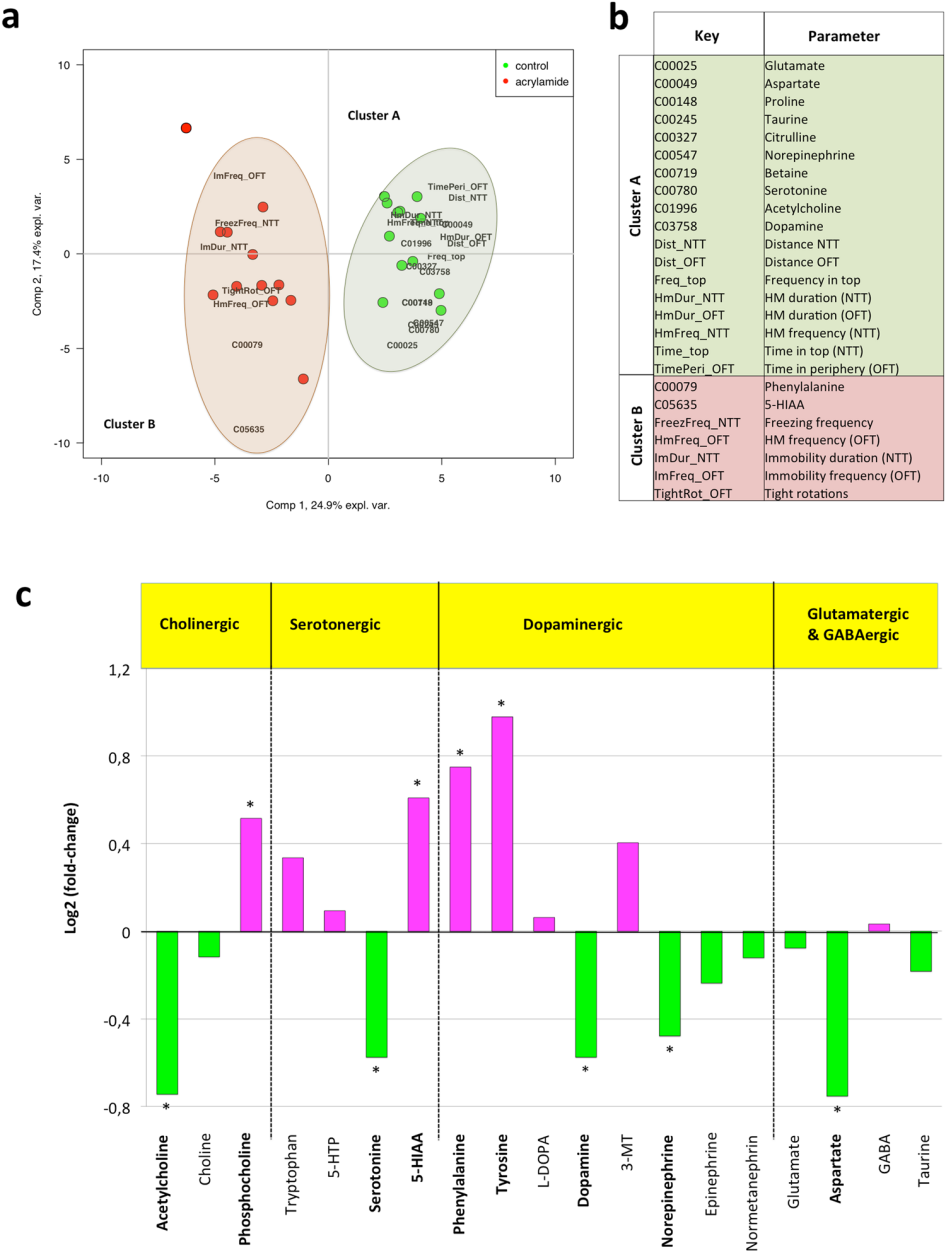
Changes in the profile of neurochemicals in the brain of adult zebrafish control and exposed to 0.75 mM acrylamide (ACR) for 3 days. (**a**,**b**) Results from the PLS-DA analysis. (**a**) Bi-plot of sample scores (circles) and parameter loadings (in black characters, only VIPs); green and red symbols correspond to control and acrylamide-treated samples. (**b**) List of parameters showing VIPs scores above 1. Green and red shadows indicate parameters associated to control and treated groups, respectively. Short (key) and complete names are indicated. (**c**) Changes in the profile of the main neurotransmitter systems in zebrafish brains exposed for 3 days to 0.75 mM ACR. Values are represented as log2 of fold change to control; green and purple columns correspond to decreased and increased levels of neurochemicals respect to the controls. Statistical analysis performed using Student’s t-test, *p < 0.05. Data from 2 independent (n = 12 per group).

A PLS-DA using both the 36 neurochemical levels (in ng/mg of brain w.w.) and 20 selected behavioral parameters correctly associated the 24 samples either to the ACR-treated or to the control groups for the two extracted components, except for single treated sample in component 1, using the 42.3% of the total variance of the parameters (Mfold test, Fig. 7a). Twenty-five out of the fifty-six parameters included in the analysis showed Variables Important for Projection (VIPs) scores above 1, twelve neurochemical levels and thirteen behavioral parameters (Figs 7b and S2). Most VIPs were associated to control samples (green areas in Fig. 7), indicating a reduction of the parameters’ values (either concentrations or frequencies) in ACR-treated animals. Most important neurotransmitters (acetylcholine, dopamine, serotonin, norepinephrine, glutamate) showed this association to non-treated samples, as did different behavioral parameters linked to locomotor activity (distance, high mobility) or exploratory behavior (time and frequency in top). Conversely, behavioral parameters related with an anxiogenic response (freezing behavior and immobility) were associated to ACR-treated samples (Fig. 7, red areas), as well as the dopamine precursor phenylalanine and the serotonin metabolite 5-HIAA.

When the main neurotransmitter systems were analyzed with more detail (Fig. 7c), a significant effect of ACR on cholinergic, serotonergic and dopaminergic systems was found. The effect of ACR on dopaminergic system is especially dramatic, with a significant increase in the levels of the precursors phenylalanine and tyrosine, and a concomitant significant decrease in the levels of the neurotransmitters dopamine and norepinephrine. A similar reduction in the content of dopamine and norepinephrine has been described in the brain of rats exposed to ACR^8^, and the depletion of these neurotransmitters for some neuroactive chemicals, as reserpine, have been related with symptoms of CNS depression, including sedative or depression-like action^8,27^. Moreover, when the levels of dopamine and norepinephrine were decreased in adult zebrafish after injection of MPTP and 6-OHDA, a phenotype characterized by hypolocomotion and increased turn angle was reported^47^. All these data suggest that the decrease in the dopamine and norepinephrine content found in the brain of ACR-treated fish might be involved in the hypolocomotion and the angular behavior exhibited by these animals. Interestingly, in addition to the hyperactivity, dorsal striatum of nice null for Adgrl3 gene contains high levels of dopamine and serotonin^37^, so the observed increase in the levels of this protein in ACR-treated proteins might be involved in the observed decrease in the levels of both neurotransmitters.

Moreover, tight circling behavior has been reported after zebrafish exposure to some antagonist of the N-methyl-D-aspartate (NMDA) receptors, including ketamine and phencyclidine^26,48,49^. Consequently, the tight circling phenotype presented by ACR-treated fish might be related with the significant decrease in glutamate and aspartate, natural agonist of the NMDA- receptors, exhibited by these animals.

When the changes in the neurochemical profile in the brain of adult zebrafish exposed for 3 days to 0.75 mM ACR were compared with the changes reported in 8 dpf zebrafish larvae exposed for 3 days to 1.0 mM ACR^15^, important differences became evident. Thus, whereas the levels of the neurotransmitters acetylcholine and norepinephrine were significantly reduced by ACR-treatment in adult brain, they were significantly increased in the whole-larvae. Moreover, whereas the levels of glutamate, aspartate and serotonin were also significantly reduced in adult zebrafish brain exposed to ACR, these neurotransmitters remained unchanged in whole-larvae. The observed differences between adult brain and whole-larvae are not surprising considering that each type of sample provides a different picture of the problem. Whereas the former provides information on changes in the a fully mature CNS, the latter integrates information not only at central and peripheral nervous systems still under development, but also from other non-neural tissue.

## Conclusions

ACR acute neurotoxicity has been characterized in adult zebrafish. This neurotoxicant not only changes the transcriptomic and proteomic profiles of the brain, but also forms adducts with selected cysteine residues of specific proteins of the brain, including relevant components of the presynaptic vesicle cycling. The depression-like behavior and the concomitant depletion in the monoamine neurotransmitters found in the ACR-treated animals suggest for the fist time that the hypolocomotion commonly observed after ACR exposure might represent a motor retardation-like phenotype associated to the depletion of monoamine neurotransmitters in the brain. Moreover, the fact that the overall effects of ACR in adult zebrafish are similar to those described in the larvae support the suitability of the zebrafish ACR acute neurotoxicity recently developed in larvae for screening of molecules with therapeutic value to treat this toxic neuropathy.

## Methods

### Animals and housing

A total of 302 adult wild-type zebrafish were obtained from Piscicultura Superior (Barcelona, Spain) and maintained in fish water [reverse-osmosis purified water containing 90 µg/ml of Instant Ocean (Aquarium Systems, Sarrebourg, France) and 0.58 mM CaSO_4_ · 2H_2_O] at 28 ± 1° in the Research and Development Centre of the Spanish Research Council (CID-CSIC) facilities under standard conditions. Aquarium lighting (650–850 lx) was provided by aquarium-mounted fluorescent light with a 12 L:12D photoperiod. They were fed twice a day with flake food (TetraMin, Tetra, Germany). All procedures were approved by the Institutional Animal Care and Use Committees at the CID-CSIC and conducted in accordance with the institutional guidelines under a license from the local government (agreement number 9027).

### Experimental procedure

ACR (CAS#79–06–1, ≥99% purity) was purchased from Sigma-Aldrich (A9099; St. Louis, MO). On the day of the experiment, a fresh stock solution (500 mM ACR) was prepared directly in fish water, and then exposure solutions were prepared by diluting the stock in fish water. Adult zebrafish (≈50:50 male:female ratio) were randomly selected from the CID-CSIC facilities and exposed for 72 h to 0.65–3.0 mM ACR (Sigma-Aldrich, St. Louis, MO), at 28.5 °C and a 12 L:12D photoperiod. Control fish were maintained in fish water under identical conditions. Although ACR concentrations remain stable in our experimental conditions for more than 5 days^15^, experimental solutions were renewed daily, 30 min after feeding. After exposure, some fish were randomly selected for behavioral testing and further whole-body cortisol and metabolomics analyses, while others were selected for transcriptomic and proteomic analyses. For sample collection, fish were euthanized by inducing hypothermic shock in ice-chilled water (2° to 4 °C), and the brains and fish whole-body were immediately collected and stored at −80 °C for further analyses.

### Concentration-response analysis for lethality

LC50 was obtained by fitting responses relative to control to the nonlinear allosteric decay regression model (see Supplementary Methods for additional details).

### Behavioral testing

All testing was performed in an isolated behavioral room at 27–28 °C. Animals (≈50:50 male:female ratio) were brought to the behavioral room one hour before testing began, to acclimate to the environment, and then, behavioral testing was performed between 10:00 and 13:00 h. All fish used in this study were experimentally naïve and all behavioral testing was performed in a blind manner, with observers unaware of the experimental group. Behavioral tests used in the present study include the novel tank test (NTT), open field test (OFT), and shoaling test. In order to reduce the number of animals needed for this study, NTT and OFT were performed following a test battery approach, and after that these animals were euthanized and brain were immediately collected and stored at −80 °C for for metabolomics analysis. The suitability of using test batteries of behavioral assays in adult zebrafish has been recently demonstrated^50^.

The NTT, used to assess locomotor activity and anxiety, was performed using an experimental setup allowing monitoring and recording 2 fish simultaneously. The NTT was performed in two experimental tanks (20 cm length, 20 cm width, 25 cm height) containing 7 L (20 cm height) fish water at 28 °C. LED backlight illumination (GP-G2, Quirumed, Spain) located behind the tank provided uniform illumination for video-recording. Control and ACR-exposed fish were tested in the standard 6-min NTT. Each trial was video-recorded (AVI format, 30 fps) with the uEye Cockpit software (version 4.90; Imaging Development Systems, Germany) controlling a GigE camera (UI-5240CP-NIR-GL, Imaging Development Systems, Germany) mounted in front of the experimental tank. In order to avoid any potential tank effect, experimental group assigned to each tank was switched between trials. After the recording was complete, the videos were analyzed by Ethovision XT 13.0 (Noldus, Wageningen, the Netherlands). First of all, the front of the tank was divided into two equal virtual zones, top and bottom (Fig. 1). Then, the total distance travelled (cm), distance travelled in the top and in the bottom (cm), latency to top (s), transitions to top, time spent in the top (s) and mobility state duration (s) were determined. Moreover, erratic movements as well as number and duration (s) of freezing bouts were manually recorded by trained observers, and ethograms were constructed in order to visualize the occurrence of these behaviors and the transitions between them, with the diameter of each circle reflecting the frequency of the behavioral activity, and the width and direction of each arrow representing the frequency of the transitions between behaviors^23^.

The zebrafish OFT was performed according to Gómez-Canela *et al*.^51^, with minor modifications. An experimental setup for monitoring and recording 2 fish simultaneously was used. The OFT was performed in circular trunked conical white plastic tanks (testing tanks; 22.5 cm lower diameter × 25.0 cm upper diameter × 26.0 cm height) containing 5 L of fish water at 28 °C. The first 6 min of the trial were video-recorded (AVI format, 30 fps) with the uEye Cockpit software (version 4.90; Imaging Development Systems, Germany) controlling the GigE cameras (UI-5240CP-NIR-GL, Imaging Development Systems, Germany) placed on top of the testing tanks. Two anti-flicker LED tubes (TUT8-ST28-NFL; AS de LED®, Valencia, Spain) mounted on both sides of the test tanks provided uniform illumination for video-recording. In order to avoid any potential tank effect, experimental group assigned to each tank was switched between trials. The recorded videos were analyzed by Ethovision XT 13.0, and the total distance travelled (cm), distance traveled in the periphery and the center (cm), time spent in the periphery and the center (s), mobility state, meandering (deg/cm), turn angle (deg) and angular velocity (deg/s) were determined. Moreover, the potential tight circling behavior was also analysed^49^ by a trained observer, recording the prevalence (%) of this behavior in each experimental group, the number of fish exhibiting “high rotation” (5 or more full rotation per trial), right-handed rotations and left-handed rotations.

The shoaling behavior was measured using the shoaling test, that consists in placing a group of fish into a novel tank and quantifying their spatial behavior and movement patterns^52^. For the shoaling test, groups of 6 zebrafish from the control and ACR groups were video-recorded for 6 min in our novel tank (Fig. 3), and analyzed using 10 screenshots made every 20 s during the last half of the observation period. A total of 30 screenshots per experimental group were used for analysis in this study. For each screenshot, distance between each fish in the group was measured by using the free-processing ImageJ software (National Institutes of Health (NIH), http://rsb.info.nih.gov/ij/). Finally, the average interfish distance (cm), farthest neighbor distance (cm) and nearest neighbor distance (cm) were calculated^23,33^.

### Whole-body cortisol assay

The cortisol extraction in whole body of adult zebrafish was performed using a modified protocol developed by Cachat *et al*.^29^. Briefly, the head from control and exposed zebrafish was removed before measuring the weight of each fish. Individual samples were then homogenized in 1 mL of ice-cold 1 × PBS buffer. Samples were transferred to glass extract-O tubes and cortisol was extracted twice with 5 mL of diethyl ether (Sigma-Aldrich, USA). After adding the ether, samples were vortexed and then centrifuged at 5000 rpm for 15 minutes at room temperature. The top organic layer containing cortisol was collected into another glass tube and the process was repeated once more. The ether was left overnight under a fume hood to evaporate. Cortisol was then reconstituted in 1 mL of 1 × PBS and left to incubate overnight at 4 °C. For quantification of the cortisol levels, a human salivary cortisol ELISA kit (Salimetrics LLC, State College, PA) was used^29,30^. ELISA plate was measured in a Synergy 2 Multi-Mode Microplate Reader (BioTek Instruments – Vermont, USA). Whole-body cortisol levels in samples were determined, according to the manufacture, using a 4-parameter non-linear regression curve fit based on the absorbances of standardized concentrations, and reported as relative concentrations, ng g^−1^ of body weight for each fish.

### Skin coloration

Changes in the skin coloration in response to ACR exposure were evaluated following standard protocols^23,53^. Briefly, we employed a standardized color rating scale (1 = pale body color; 2 = dark body color) assessed visually by 3 highly-trained observers (blinded to the treatments). Coloration of adult zebrafish control (n = 18) and exposed to 0.75 mM ACR for 3 days (n = 16) was recorded, and the decision for each animal was based on consensus among all observers.

### RNA preparation and qRT-PCR analysis

Total RNA was extracted from the control (n = 17) and ACR-treated (n = 14) zebrafish brains and the expression of the nine selected genes (*gap43*, *gfap*, *mbp*, *nsfa*, *tuba1b*, *syn2a*, *syt1a*, *sytxbp1b*, *c-fos*) was determined by Real Time PCR, following standard protocols^15^ (see Supplementary Methods for additional details).

### Proteomic Analysis

Protein fraction were extracted from pools of 3 brains from control and ACR-treated adult zebrafish (5 pools/group), trypsinized and analyzed by LC-MS/MS using a Q-Exactive-Plus mass spectrometer fitted with a capillary HPLC, following standard protocols (see Supplementary Methods for additional details).

### Proteomic data Analysis

The mass spectrometry data were analyzed using the MaxQuant software 1.5.2.8 (www.maxquant.org)^54^ fitted with the Andromeda search engine^55^, searching against the *Danio rerio* Uniprot database (of March 2017 containing 59,064 entries) with mass tolerance of 20 ppm for the precursor masses and the fragment ions. Oxidation on methionine, propionamide on cysteine, histidine and lysine were accepted as variable modifications and carbamidomethyl on cysteine was accepted as fixed modifications. Minimal peptide length was set to six amino acids and a maximum of two miscleavages was allowed. Peptide and protein level false discovery rates (FDRs) were filtered to 1% using the target-decoy strategy. The identified protein table was filtered to remove the identifications from the reverse database, the common contaminants and single peptide identifications.

Data were quantified by normalized label free analysis using the same MaxQuant software (LFQ intensities), based on extracted ion currents (XICs) of peptides enabling quantitation from each LC/MS run for each peptide identified in any experiment.

Statistical analysis of the identification and quantitation results was performed using Perseus software 1.5.1.6^56^. Student’s T-test was done with 0.05 FDR and 250 randomizations. Proteins with P value less than 0.05 and a difference of at least 2 fold between groups were labeled as differential.

### Analysis of neurochemicals by LC-MS/MS

Individual zebrafish adult brain were extracted using a previous published method^12^. In the present study, 38 neurochemicals, including neurotransmitters, precursors, metabolites and neuromodulators were determined by liquid chromatography coupled to tandem mass spectrometry (LC-MS/MS) using a Synergi Polar-RP 80 Å column (250 mm × 4.6 mm i.d., particle size 4 μM, Phenomenex, Torrance, USA; see Supplementary Methods for additional details).

### Data analysis

Data were analyzed with IBM SPSS 19.0 (Statistical Package 2010, Chicago, IL), using chi-square test, Student’s t-test or one-way ANOVA followed by Dunnett’s multiple comparison test. Data are presented as the mean ± SEM of 2–3 independent experiments, unless otherwise stated. Significance was set at P < 0.05. Analysis of the qRT-PCR data, which was normally distributed (Levene’s test), was performed using the ΔΔCt method. Differences between the control and treated groups were analyzed by Student’s t-test.

Partial least squares-discriminant analysis (PLS-DA) were performed to determine the predictive capacity of combined behavior and chemical analysis data for discriminating between control and treated samples, using the package “mixOmics” in the “R” environment^57^. Missing values (less than 5%) were replaced by averaged values for each variable; untransformed values were used for all the analyses. Variables Important for Projection (VIPs) and PLS-DA performance (multiple-fold cross-validation, or Mfold) were calculated using the package “RVAideMemoire”, also in R.

### Data availability

The mass spectrometry proteomics data have been deposited to the ProteomeXchange Consortium via the PRIDE partner repository with the dataset identifier PXD008993. The authors declare that all other data supporting the findings of this study are available within the manuscript and its Supplementary Information files, or are available from the corresponding author upon request.

## Electronic supplementary material

Supplementary Information

Supplementary Video S1

Supplementary Video S2

Supplementary Video S3

Supplementary Video S4

Supplementary Dataset1

Supplementary Dataset2

Supplementary Dataset3

Supplementary Dataset4
