# Supplementary material for: Acrylamide acute neurotoxicity in adult zebrafish
Source: Sci Rep. 2018 May 21;8:7918. doi: 10.1038/s41598-018-26343-2 (PMC5962567; doi:10.1038/s41598-018-26343-2)
Supplement: Supplementary file 1 — Supplementary Information [file 41598_2018_26343_MOESM1_ESM.pdf]

Supplementary Information

Acrylamide acute neurotoxicity in adult zebrafish

Melissa Faria, Tamar Ziv, Cristian Gómez-Canela, Shani Ben-Lulu, Eva Prats, Karen Adriana Novoa-Luna, Arie Admon, Benjamin Piña, Romà Tauler, Leobardo Manuel Gómez-Oliván, Demetrio Raldúa*.

*Address correspondence to: Demetrio Raldúa, Institute of Environmental Assessment and Water Research (IDAEA-CSIC), Jordi Girona 18, 08034 Barcelona, Spain. Telephone: +34-93-4006138. E-mail: drpqam@cid.csic.es

Title of file for HTML: Supplementary Information
Description: Supplementary Methods, Supplementary Figures, Supplementary Tables, Supplementary References.

Title of file for HTML: Supplementary Dataset 1
Description: **List of all the zebrafish proteins identified by µLC-MS/MS in at least 3 of the samples and with at least 2 different tryptic peptides in the brain of control (Con) and ACR-treated (0.75 mM ACR) adult zebrafish.**

Title of file for HTML: Supplementary Dataset 2
Description: **List of all the zebrafish proteins differentially expressed in the brain of control (Con) and ACR-treated (0.75 mM ACR) adult zebrafish.**

Title of file for HTML: Supplementary Dataset 3
Description: **List of all the zebrafish proteins with covalent modifications on specific cysteine residues in at least 3 of the ACR-brains analyzed.**

Title of file for HTML: Supplementary Dataset 4
Description: **List of all the zebrafish proteins with covalent modifications on specific cysteine residues present in both the whole-larvae and the brains after 3 days exposure to 1 mM ACR (whole-larvae) or 0.75 mM ACR (adult zebrafish).**

Title of file for HTML: Supplementary Video S1
Description: **Representative behaviour of the control and ACR-treated zebrafish in the novel tank test .**

Title of file for HTML: Supplementary Video S2
Description: **Freezing bout in a representative ACR-treated zebrafish.**

Title of file for HTML: Supplementary Video S3
Description: **Representative swimming behaviour in a control zebrafish and droopy tail behaviour in ACR-treated zebrafish.**

Title of file for HTML: Supplementary Video S4
Description: **Differences in the swimming between representatives one control and one ACR-treated zebrafish exhibiting tight circling.**

Supplementary Information

Supplementary Methods

Concentration-response analysis for lethality

LC50 values were obtained by fitting responses relative to control treatments (R) to the nonlinear allosteric decay regression model depicted in Eq. (1):

$$R(Ci) = \frac{1}{1 + \frac{C_i k}{EC/LC50k}}$$

where

$R(C_i)$ proportional biological response at concentration C_i relative to controls

C_i concentration of the toxic substance (i)

LC50 the half saturation constant (concentration of ACR that caused 50% mortality of larvae)

k decay index

The allosteric decay model was selected to fit the obtained data because it can describe nonlinear type responses. Model accuracy was assessed using the adjusted coefficient of determination (r^2) and analysing residual distribution. The significance of the entire regression and regression coefficients were determined by analysis of variance (ANOVA) and Student’s t-test, respectively.

All analyses were conducted using statistical analysis software (IBM SPSS 19.0 and SigmaPlot 11.0, 2008, Systat Software Inc.).

RNA preparation and qRT-PCR analysis.

Total RNA was extracted from individual brains from 17 control and 14 ACR-treated adult zebrafish, from 3 independent experiments, using Trizol Reagent (Invitrogen Life Technologies, Carlsbad, CA). RNA concentration was measured by spectrophotometric absorption in a NanoDrop ND-8000 spectrophotometer (NanoDrop Technologies). After DNaseI treatment (Ambion, Austin, TX), total RNA was retro-transcribed to cDNA with First Strand cDNA synthesis Kit (Roche Diagnostics, Mannheim, Germany) according to manufacturer's instructions. Real Time PCR was performed in LightCycler ® 480 Real-Time PCR System (Roche Diagnostics, Mannheim, Germany) using SYBR Green PCR Master Mix (Roche Diagnostics, Mannheim, Germany). Cycling parameters were 95ºC for 15 min followed by 45 cycles of 95ºC for 10 s and 60ºC for 30 s. Three technical replicates were run for each sample. Appropriate primers for the nine selected genes (*gap43*, *gfap*, *mbp*, *nsfa*, *tuba1b*, *syn2a*, *syt1a*, *sytxbp1b, c-fos*) were designed using Primer Express 2.0 software (Applied Biosystems, Foster City, CA) and the Primer-Blast server (http://www.ncbi.nlm.nih.gov/tools/primer-blast; primer sequences in Table S1) and synthesized by Sigma. The house-keeping genes *ppia2* and e*ef1a* were selected as reference genes[1].

Relative mRNA abundances of different genes were calculated from the second derivative maximum of their respective amplification curves (Cp, calculated by triplicates). To minimize errors on RNA quantification among different samples,

Cp values for target genes (Cptg) were normalized to the average Cp values for ppia2, used as reference gene, following Eq. (2)

$\Delta Cptg = Cpppia2 - Cptg$

Changes in mRNA abundance in samples from different treatments were calculated by the ΔΔCp method[2], using corrected Cp values from treated and non-treated samples (Eq. (3))

$\Delta\Delta Cptg = \Delta Cptg\_untreated - \Delta Cptg\_treated$

Fold-change ratios were derived from those values.

Proteomic Analysis.

Proteolysis

The protein fractions were extracted with 9 M Urea, 400 mM ammonium bicarbonate and 10 mM DTT. 20 μg protein from each sample were reduced with 2.8 mM DTT (60°C for 30 min), modified with 8.8 mM iodoacetamide in 400 mM ammonium bicarbonate (in the dark, room temperature for 30 min) and digested in 2M urea, 100 mM ammonium bicarbonate with modified trypsin (Promega) at a 1:50 enzyme-to-substrate ratio, overnight at 37°C. An additional second trypsinization was done for 4 hours.

Mass spectrometry analysis

The tryptic peptides were desalted using C18 tips (Harvard) dried and re-suspended in 0.1% Formic acid. The peptides were resolved by reverse-phase chromatography on 0.075 X 180-mm fused silica capillaries (J&W) packed with Reprosil reversed phase material, as in Ishihama et al.[3]. The peptides were

eluted with linear 180 minutes gradient of 5 to 28% 15 minutes gradient of 28 to 95% and 25 minutes at 95% acetonitrile with 0.1% formic acid in water at flow rates of 0.15 μl/min. Mass spectrometry was performed by Q Exactive plus mass spectrometer (Thermo) in a positive mode using repetitively full MS scan followed by collision induces dissociation (HCD) of the 10 most dominant ions selected from the first MS scan.

The mass spectrometry data from all the biological repeats was analyzed using the MaxQuant software 1.5.2.8[4] vs. the *Danio rerio* section of the Uniprot database with 1% FDR. The data was quantified by label free analysis using the same software. Oxidation on methionine, propionamide and carbamidomethyl on cysteine, propionamide on histidine and lysine were accepted as variable modifications. Minimal peptide length was set to six amino acids and a maximum of two miscleavages was allowed. The identified protein table was filtered to remove the identifications from the reverse database, the common contaminants and single peptide identifications.

T test was done between the groups (using Perseus software) with 0.05 permutated FDR and 250 randomizations. Differential proteins were proteins identified with at least 2 evidences, at least in 3 samples with P value of 0.05 or less and log2 difference between the groups of 0.8 or more.

Statistical analysis

Statistical analysis of the identification and quantization results was done using Perseus software 1.5.2.4[5]. Proteins with P value less than 0.05 and a difference of at least 2 fold between groups were labelled as differential.

Analysis of neurochemicals by LC-MS/MS.

Zebrafish adult brains were extracted by a method adapted from Gomez-Canela et al.[6,7]. Samples were spiked with 500 ng of isotope labeled solution of L-aspartic acid-^{15}N (internal standard) and then, 500 µL of MeOH:H_2O (90:10) were added to each pool and shaken. Three stainless steel beads (3 mm diameter) were placed in each sample and were homogenized using a bead mill homogenizer (TissueLyser LT, Qiagen) at 50 oscillations per min during 90 s. After this, samples were centrifuged for 20 min at 13,000 rpm at 4ºC. Finally, the supernatant was filtered using 0.20 µM PTFE filters (DISMIC -13 JP, Advantec®) and stored at -80 ºC until liquid chromatography coupled to tandem mass spectrometry (LC-MS/MS) analysis. Neurotransmitters were measured using LC connected to a triple quadrupole detector (Xevo TQD, Waters, USA) (LC-MS/MS). A Synergi Polar-RP 80 Å column (250mm x 4.6 mm ID, particle size 4 µM, Phenomenex, Torrance, USA) was used to separate the target compounds. The mobile phase composition consisted of binary mixtures with 0.1% of formic acid in water (A) and 0.1% formic acid in MeOH (B). Gradient elution started at 95% A and 5% B in the first 2 min and increased to 30% B in 5 min. Then, gradient increased to 95% B in the next 13 min, and held for 5 min. Initial conditions to stabilize the system were attained in 5 min being 30 min the total run time. The flow rate was set at 600 µL min^{-1} and 10 µL were injected. Neurotransmitters were measured under positive electrospray ionization (ESI+). Finally, to identify each compound, acquisition was performed in selected reaction monitoring (SRM) mode using two transitions from precursor ion to product ions. Supplementary Table S3 displays the SRM transitions as well as cone voltages and collision energies used to identify the target compounds.

More details about the chromatographic and mass spectral conditions are given in a previous published paper[7]. Experimental data were acquired and processed using the MassLynx v4.1 software package.

Supplementary Figures

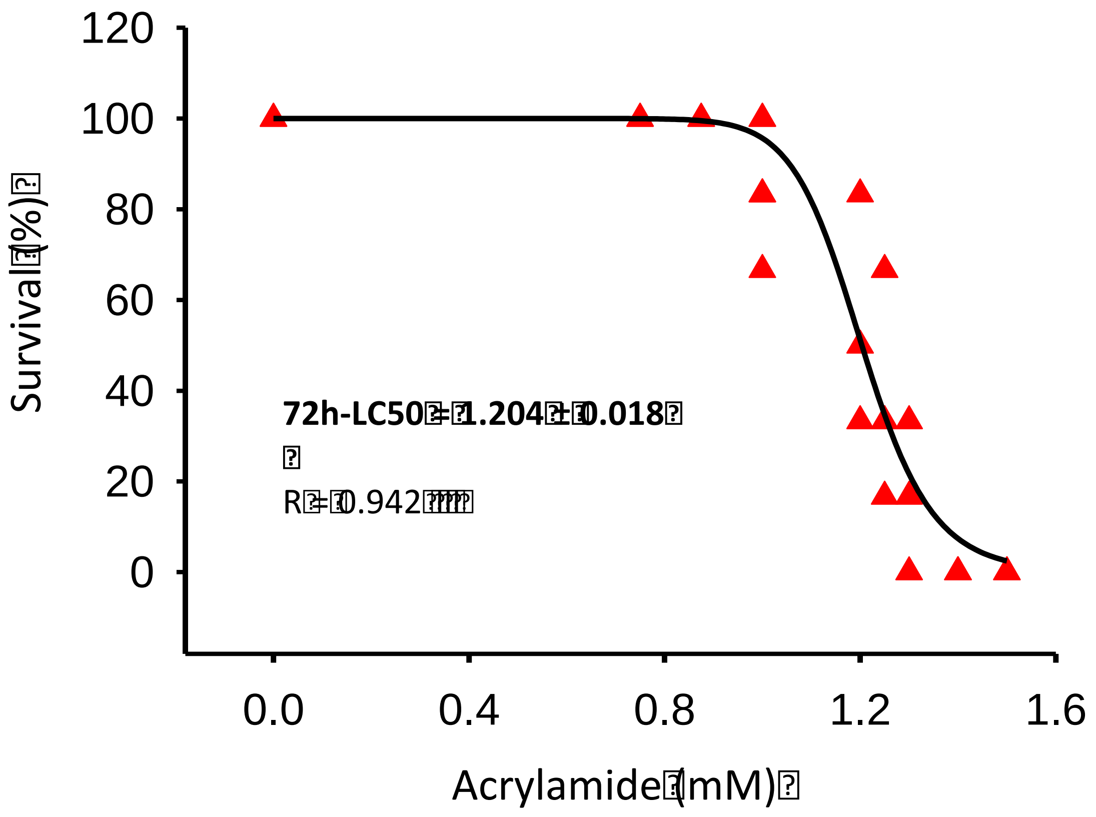

Supplementary Figure S1: Concentration-response analysis of the effect of ACR on the survival of adult zebrafish. Regression parameters according to Eq.1 of fitted models to mortality responses were: LC50: 1.204 mM ACR; / SEM: 0.02/ P < 0.0001/ r^2: 0.8818/ n= 123. Abbreviations: ACR, acrylamide.

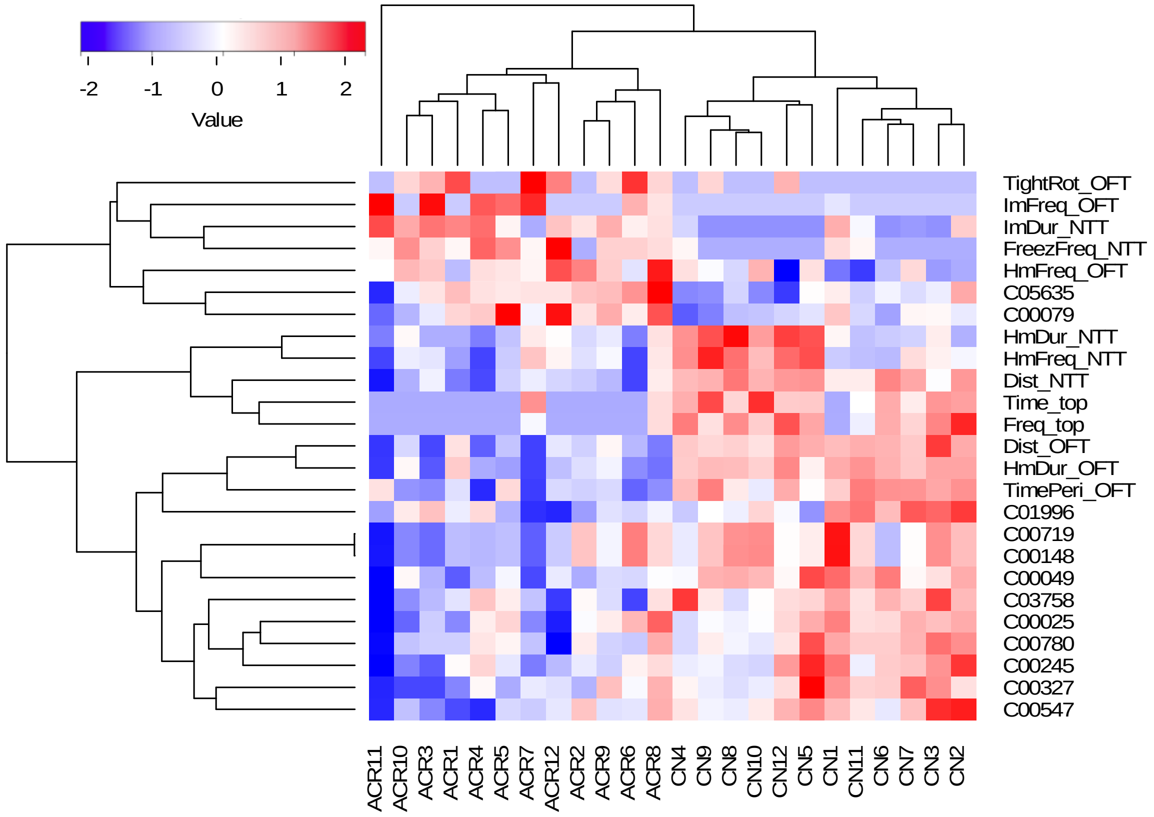

Supplementary Figure S2. Hyerarchical clustering of brain samples by their relative levels in the 25 parameters (chemical or behavioral) showing VIPs scores above 1 in the PLS DA. Treated and control samples are labeled as "ACR" and "CN", respectively. Blue and red squares indicate low and high levels of each parameter (rows) in a given sample (columns). The heat map was calculated using row-standardized data. Please refer to main text' Figure 5 for parameter description.

Supplementary Table S1. Primers used for qPCR, including ZFIN and GeneBank accession numbers.

Gene	ZFIN	GeneBank	Gene description	Sequence		Amplicon
gfap	ZDB-GENE-990914-3	NM_131373	glial fibrillary acidic protein	FW<br>RV	5'-GGATGCAGCCAATCGTAAT<br>5'-TTCCAGGTCACAGGTCAG	97 bp
mbp	ZDB-GENE-030128-2	AY860977	myelin basic protein a	FW<br>RV	5'-AATCAGCAGGTTCTTCGGAGGAGA<br>5'-AAGAAATGCACGACAGGGTTGACG	102 bp
gap43	ZDB-GENE-990415-87	NM_131341.1	growth associated protein 43	FW<br>RV	5'-CAGCCGACGTGCCTGAA<br>5'-GGATTCCTCAGCAGCGTCTG	71 bp
syn2a	ZDB-GENE-040718-341	NM_001002597	synapsin IIa	FW<br>RV	5'-GTGACCATGCCAGCATTTC<br>5'-TGGTTCTCCACTTTCACCTT	80 bp
tuba1b	ZDB-GENE-030822-1	NM_194388.2	tubulin, alpha 1b	FW<br>RV	5'-AATCACCAATGCTTGCTTCGAGCC<br>5'-TTCACGTCTTTGGGTACCACG	117 bp
nsfa	ZDB-GENE-030616-37	NM_001044328.1	N-ethylmaleimide-sensitive factor a	FW<br>RV	5'-CGCGGCTTCTTCGAGTAACA<br>5'-GAAGTGTGATCTCCGTCAGGTT	134 bp
syt1a	ZDB-GENE-040718-165	NM_001327829	synaptotagmin Ia	FW<br>RV	5'-AAAGGGAAGAGACGGCTGTG<br>5'-GGAGCCAGGCAGAAGCTTTA	130 bp
stxbp1b	ZDB-GENE-060531-166	NM_001089376.1	syntaxin binding protein 1b	FW<br>RV	5'-ACGCTGAAAGAGTACCCAGC<br>5'-CTCCCAAAGTGGGGTCATCC	118 bp
c-fos	ZDB-GENE-031222-4	NM_205569.1	c-fos	FW<br>RV	5'-TGCAGCACGGCTTCACCGAG<br>5'-CGGGCATGAAGAGATCGCCGT	129 bp
ppia2	ZDB-GENE-030131-8556	NM_212758,1	2-peptidylpropyl isomerase A	FW<br>RV	5'-GGGTGGTAATGGAGCTGAGA<br>5'-AATGGACTTGCCACCAGTTC	179
eef1a1	ZDB-GENE-990415-52	NM_131263,1	eukaryotic translation elongation factor 1 alpha 1	FW<br>RV	5'-CGTCTGCCACTTCAGGATGTG<br>5'-ACTTGCAGGCGATGTGAGCAG	376 bp

Supplementary Table S2. Levels (ng/mg f.w.) of the main neurotransmitters, precursors and degradation products in the brain of adult zebrafish control and exposed to 0.75 mM ACR for 72 h.

	**Control**	**0,75 mM ACR**	***P* value**[a]
**Acetylcholine**	2.43 ± 0.27	1.51 ± 0.28	**0.027**
**Phosphocholine**	49.43 ± 3.25	70.90 ± 5.52	**0.003**
**Choline**	48.08 ± 3.40	49.11 ± 6.83	0.695
**Tryptophan**	5.52 ± 0.28	6.96 ± 0.64	0.051
**5-HTP**	0.054 ± 0.043	0.012 ± 0.001	0.337
**Serotonin**	0.95 ± 0.05	0.74 ± 0.06	**0.014**
**5-HIAA**	0.2 ± 0.01	0.30 ± 0.02	**7.98 x 10^{-5}**
**Phenylalanine**	8.51 ± 0.46	14.31 ± 1.41	**0,001**
**Tyrosine**	11.79 ± 2.74	22.86 ± 3.18	**0,015**
**L-DOPA**	0.23 ± 0.07	0.22 ±0.11	0.943
**Dopamine**	0,41 ± 0.04	0.28 ± 0.03	**0,010**
**3-Methoxytyramine**	0,016 ± 0.003	0.022 ± 0.004	0.321
**Norepinephrine**	1.90 ± 0.13	1.38 ± 0.13	**0.008**
**Epinephrine**	2.38 ± 0.56	2.03 ± 0.26	0.582
**Glutamate**	617.77 ± 20.20	590.32 ± 24.01	0.391
**Aspartate**	50.77 ± 2.56	29.46 ± 2.22	**1.12 x x 10^{-5}**
**GABA**	304.17 ± 8.78	314.86 ± 14.33	0.531

[a] *P* value from Student's-t test

Supplementary References

1 Oliveira, E., *et al*. Retinoic acid receptors' expression and function during zebrafish early development. *J Steroid Biochem Biol.* **138**, 143-151 (2013).

2 Pfaffl, M. W. A new mathematical model for relative quantification in real-time RT–PCR. *Nucleic Acids Res.* **29**, e45-e45 (2001).

3 Ishihama, Y., Rappsilber, J., Andersen, J. S., Mann, M. Microcolumns with self-assembled particle frits for proteomics. *J Chromatograph A.* **979**, 233-239 (2002).

4 Cox, J. & Mann, M. MaxQuant enables high peptide identification rates, individualized ppb-range mass accuracies and proteome-wide protein quantification. *Nat Biotechnol.* **26**, 1367–1372 (2008).

5 Tyanova, S. et al. The Perseus computational platform for comprehensive analysis of (prote) omics data. *Nat Methods.* **13**, 731–740 (2016).

6 Gómez-Canela, C., Prats, E., Piña, B. & Tauler, R. Assessment of chlorpyrifos toxic effects in zebrafish (Danio rerio) metabolism. *Environ Pollut.* **220**, 1231-1243 (2017).

7 Gómez-Canela, C. *et al.* Comprehensive characterization of neurochemicals in three zebrafish chemical models of human acute organophosphorus poisoning using liquid chromatography-tandem mass spectrometry. *Anal Bioanal Chem.* **410**, 1-14 (2018).
